# Development of Photovoltaic Module with Fabricated and Evaluated Novel Backsheet-Based Biocomposite Materials

**DOI:** 10.3390/ma12183007

**Published:** 2019-09-17

**Authors:** M. H. Alaaeddin, S. M. Sapuan, M. Y. M. Zuhri, E. S. Zainudin, Faris M. AL-Oqla

**Affiliations:** 1 Advanced Engineering Materials and Composites Research Center, Department of Mechanical and Manufacturing Engineering, Faculty of Engineering, Universiti Putra Malaysia, Serdang 43400 UPM, Selangor, Malaysia; 2 Laboratory of Bio-Composite Technology, Institute of Tropical Forestry and Forest Products, Universiti Putra Malaysia, Serdang 43400 UPM, Selangor, Malaysia; 3 Department of Mechanical Engineering, Faculty of Engineering, Hashemite University, Zarqa 13133, Jordan

**Keywords:** solar modules, photovoltaic applications, novel backsheets, electrical and thermal performances

## Abstract

Photovoltaic backsheets have considerable impact on the collective performance of solar cells. Material components should withstand certain temperatures and loads while maintaining high thermal stability under various weather conditions. Solar modules must demonstrate increased reliability, adequate performance, safety, and durability throughout the course of their lifetime. This work presents a novel solar module. The module consists of an innovative polyvinylidene fluoride-short sugar palm fiber (PVDF-SSPF) composite backsheet within its structure. It was electrically and thermally evaluated. The current-voltage characteristics (I-V) were obtained using the solar module analyzer, PROVA 210PV. A thermal evaluation was accomplished using a temperature device, SDL200. The thermal test consisted of two different assessments. The first targeted the surface and backsheet of the developed module to correlate their performance from within. The second assessment compared the thermal performance of the fabricated backsheet with the conventional one. Both tests were combined into a heatmap analysis to further understand the thermal performance. Results revealed that the developed module exhibited reasonable electrical efficiency, achieving appropriate and balanced I-V curves. PVDF-SSPF backsheets proved to be thermally stable by displaying less heat absorbance and better temperature shifts. Additional research efforts are highly encouraged to investigate other characteristics. To enhance performance, further analyses are needed such as the damp heat analysis, accelerated aging analysis, and heat dissipation phenomena.

## 1. Introduction

Photovoltaic (PV) backsheets are critical components of solar modules. Appropriate selection of materials is required for modules to exhibit higher performance and reliable results [1]. PV module backsheets are exposed to aggressive field environments such as mechanical loads, moisture, and combined temperature cycles. Such exposure may cause debonding, resulting in module degradation and loss of function [2]. The most important indicator of durability, reliability, and safety of PV modules is their performance. This strongly relies on component materials that perform under various stresses in outdoor environments [3]. PV backsheets are multilayers composed of various polymeric materials and inorganic modifiers. These multilayer structures support their thermo-mechanical, electrical, optical, and barrier properties. Backsheet components include fluoropolymers and thermoplastics, such as polyvinyl fluoride (PVF), which provide outstanding weatherability. Polyvinylidene fluoride (PVDF), polyesters (PET), polyamides (PA), ethylene chlorotrifluoroethylene (ECTFE), ethylene vinyl acetate copolymer (EVA), and tetrafluoroethylene hexafluoropropylene vinylidene fluoride (THV) are some examples [4]. PVDF is a thermoplastic polymer known for its high impact strength, altered density, solubility, thermal expansion, hardness, and degree of crystallinity. It has superior pyroelectric and piezoelectric coefficients. It is a strong candidate for various indoor and outdoor applications such as PV applications, encapsulations, insulations, membranes, laminations, coatings, sensing materials, aircraft interiors, composites structures, and biomaterials [5]. PVDF was previously combined with short sugar palm fiber (SSPF). The biocomposite revealed outstanding mechanical, nanomechanical, thermal, optical, physical, and technical properties. These assets make it highly suitable for PV applications [6,7]. The introduction of innovative backsheets will reduce the complete reliance on polymeric and inorganic materials that are detrimental to the environment. Natural fiber composites (NFCs) have recently become the subject of interest. The use of such composites may open new horizons since they are significant alternatives to harmful synthetic fibers. They maintain outstanding properties for various industrial and engineering applications and can withstand advanced conditions. Natural fibers reinforced with polymers and conductive polymers present new composites that can serve as alternatives to synthetic materials. Conductive polymers exhibited compelling evidence of having eminent control over electrical stimuli. They possess outstanding properties compared to other electroactive biomaterials; i.e., electrets, piezoelectric, and photovoltaic materials [8]. Short natural fibers can adjust the dielectric response of designated polymeric matrices. They are suitable for predicting various factors that influence the characterization of NFCs. This can help determine their electrical, biological, and mechanical properties [9]. This work introduces a newly fabricated PV module combined with novel PVDF-SSPF backsheets. An evaluation was conducted to assess the module’s functionality and to understand the complex behavior of composites under various weather conditions and working arrangements.

## 2. Materials and Methods

### 2.1. Module Development

The solar module’s development process adhered to certain findings. The main development procedure was to replace a commercial backsheet with the novel backsheet structure consisting of polyvinylidene fluoride (PVDF) and short sugar palm fiber (SSPF) composites. Commercial backsheets suffer from multiple failure modes such as delamination, yellowing, hydrolysis failures, poor adhesion, thermal instability, heat transfer, and heat absorbance issues [10,11,12].

PVDF-SSPF backsheet composites were employed as new materials for enhancing the module’s thermal stability, durability, and overall performance under various weather conditions. PVDF-SSPF composites exhibited outstanding mechanical, nanomechanical, thermal, optical, physical, and technical properties [6,7]. They can be utilized as possible materials for increasing durability and thermal stability in solar modules [6]. The results encourage the use of composites as backsheets in photovoltaic applications.

**Figure 1:**
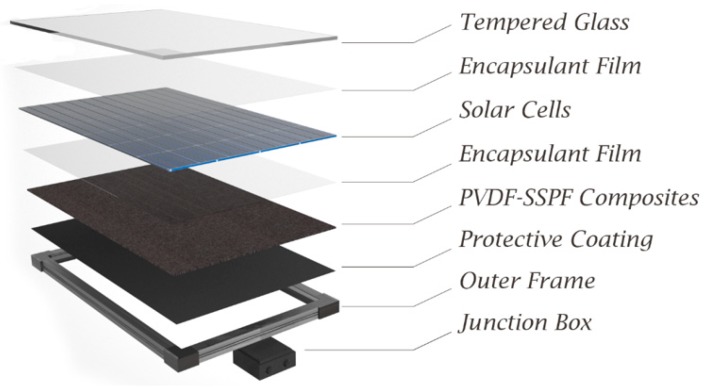
A schematic diagram of the proposed solar module including utilized components and functional parts.

The solar module was developed at the Institute of Advanced Technology (ITMA), Advanced Engineering Materials, and Composites Research Center (AEMCRC). It was evaluated at the Hybrid AgriVoltaic System (HAVs), Universiti Putra Malaysia (UPM). Efficient polycrystalline solar cells were used to generate electricity in the solar module. The module had 36 solar cells, and each cell was the size of 78 mm by 52 mm. Each solar cell provides 0.55 W (Pmax), 0.47 V (Vmp), 0.6 V (Voc), and 0.032 (Imp). The backsheets were fabricated using polyvinylidene fluoride (PVDF) and short sugar palm fiber (SSPF) composites. The main components of the module were solar cells, PVDF-SSPF composites, tempered glass, encapsulant films, and backsheet sealant. Tempered glass protected the surface of solar cells and the module from possible damage that may lead to module failure. It strengthened the surface area and prevented solar cells from becoming impaired when applying load or tension on the solar module. The tempered glass was used as the first layer over the encapsulant and solar cells layer. A protective layer was placed between the solar cells and PVDF-SSPF backsheet composites. The second encapsulation film was optional since the composite was waterproof. It was sufficiently dispersed to adequately protect the solar cells. Figure 1 provides a schematic diagram of the fabricated solar module consisting of PVDF-SSPF backsheet composites.

To fabricate the backsheet composites, polyvinylidene fluoride (PVDF) was obtained and reinforced with short sugar palm fiber (SSPF). The fiber was treated prior to composition arrangement. The obtained SSPF was further prepared and mixed with PVDF using a Brabender measuring mixer, model: W 50 EHT (Brabender, Duisburg, Germany). The loading of fiber into the polymer matrix was 30%. This was recommended by numerous works. The amount of fiber was determined to ensure outstanding environmental, mechanical, technical, and physical properties. This will lead to adequate reinforcement in composites [6,7,13,14,15,16]. The composites underwent hot and cold pressings at a maximum pressure of 160 Bar. This was accomplished in three successive phases: preheat, hot press, and cold press. The dimensions of the obtained backsheet composites were 530 mm (L) × 360 mm (W) × 1 mm (T). The backsheet was joined to the module after testing and passing the electrical evaluation. A thin layer of black sealant was added following the attachment of the backsheet composite. This was a precautionary measure to ensure better thermal evaluation, and to acquire additional information on heat dissipation phenomena.

### 2.2. Electrical Testing of Solar Module

The solar module analyzer, PROVA 210PV (PROVA INSTRUMENTS INC., New Taipei, Taiwan), was used to evaluate the module’s performance. The device’s auto-scan of 60 V and 12 A provides I-V curves or current-voltage characteristic curves, efficiency percentage evaluation for solar module performance, and maximum module power analysis. Key parameters such as the cell’s maximum power output (Pmax), current at Pmax (Imax), voltage at Pmax (Vmax), conversion efficiency of the module (η), open-circuit voltage (Voc), short-circuit current (Isc), and fill factor (FF) are important measurements to further identify possible improvements in module performance [17]. Cell series resistance, cell diode properties, and cell shunt resistance are also significant determinants of solar cell performance. The analyzed data can be utilized to improve PV system performance. The best structural designs for efficient energy harnessing can be identified using field verification data for better evaluation. The electrical assessment of a solar module helps determine its efficiency and provides data analysis on effective energy harnessing. The electrical performance of the solar module was observed and monitored during humid, rain, sunny, and cloudy weather conditions. The I-V curves were taken for each evaluation round throughout the testing procedure. Furthermore, a tripartite analysis for the Vopen-(V), Ishort-(A) and Pmax-(W) was also carried out under random sampling, the analysis provides partial evaluation of the electrical performance of the developed solar module. In the tripartite analysis, four I-V samples were designated, each consists of eight successive readings.

### 2.3. Thermal Testing of Solar Module

**Figure 2:**
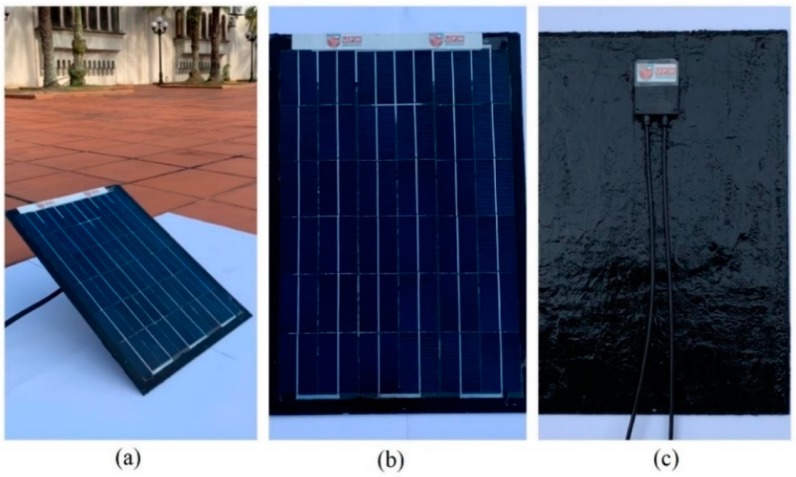
Developed solar module: (**a**) module while exposed to sun radiation; (**b**) front view of the module; (**c**) back view of the module showing the sealed backsheets.

The temperature meter, four-channel SDL200, provided by (EXTECH, Boston, MA, USA) was employed to measure the temperature of the developed solar module and its respective backsheet. Thermal testing was divided into two different analyses. The first thermal analysis identified two logs to measure the temperature of the surface of the developed solar module. The second thermal analysis employed one log to comparatively measure the temperature of PVDF-SSPF backsheet composites attached to the developed module. A comparison between the backsheet’s thermal performance in the developed module and that of the conventional module was included using similar parameters and weather conditions. The analysis was accomplished to further understand the thermal behavior of the developed module using PVDF-SSPF composites against conventional backsheet technology. Conventional backsheets are usually comprised of materials such as polyester, polymeric white sheets, and transparent laminates. Thermal efficiency in solar modules are of paramount importance to sufficiently understand their behavior. Better characterization can be accomplished to improve the modules’ efficiency. Solar modules encounter efficiency problems when exposed to excessive heat. Such temperatures cause degradation and failure modes. New solutions are needed to maintain better thermal stability. The effect of solar cells’ temperature on the solar module’s overall performance and lifespan remains one of the major drawbacks in such systems [18]. Hence, it is useful to determine the impact of temperature and irradiance on solar cells. This requires maintaining proper efficiency measurements which must be correlated with solar parameters. A change in parameters can affect the efficiency of the employed modules. To better understand the thermal behavior of the solar module, a heatmap analysis showing the correlations and thermal performance of module’s surface and backsheet temperatures is provided. The heatmap analysis also depicts a comparison between the PVDF-SSPF backsheet composites and the conventional backsheets regarding their thermal performance. The developed module is shown in Figure 2.

The solar module was tested before and after attaching the backsheets. Previous tests were accomplished to identify faults or wiring issues within the module. The final test was accomplished under various climates: normal, rain, hot, and humid weather conditions. To evaluate a solar module, the electrical characteristics considered are the maximum power, maximum power current, maximum power voltage, percent of tolerance rated value, maximum system voltage, open-circuit voltage (Voc), and short-circuit current (Isc). Determining solar module performance is not only dependent on basic characteristics. Important issues must be considered during evaluation such as the environment where the module is being placed, ambient temperature, wind velocity, and humidity [19]. Assessing solar cell performance involves measurements such as voltage, intensity, temperature, radiation spectrum, and wind speed. The solar conversion efficiency *η* is the prominent parameter. It is defined as the maximum electrical power (Pmax) produced by a solar cell divided by the incident photon power (Pin). Increase in the solar module’s ambient temperature may cause deficiency in supplying the expected energy. The phenomena occurs under hot weather conditions [20]. Temperature represents a significant factor that influences the solar module and its degradation process [21].

## 3. Results and Discussion

### 3.1. Electrical Performance of Solar Module

**Figure 3:**
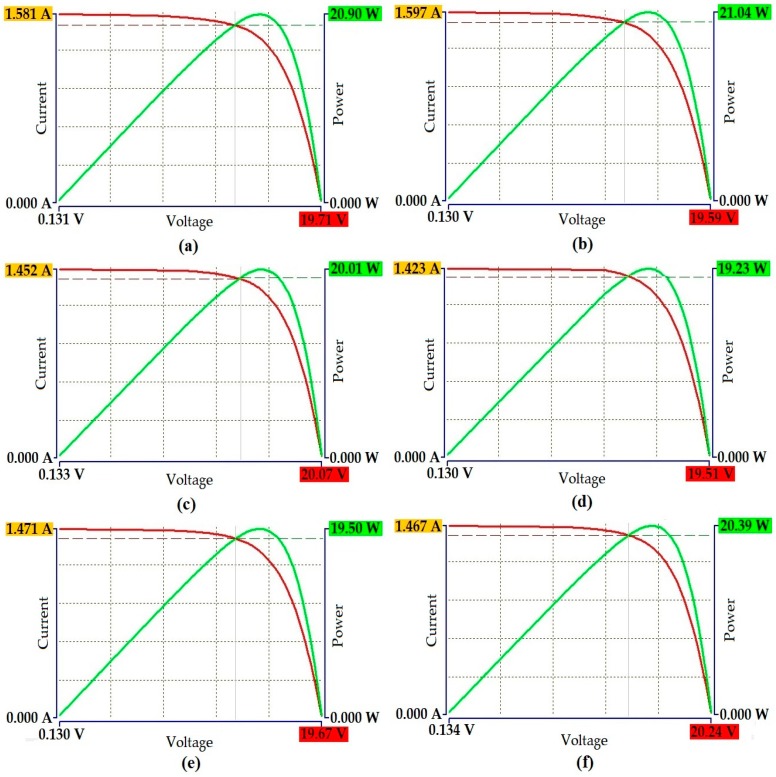
I-V curves generated by developed module: (**a**) 20.90 W achievement; (**b**) 21.04 W achievement; (**c**) 20.01 W achievement; (**d**) 19.23 W achievement; (**e**) 19.50 W achievement; and (**f**) 20.39 W achievement.

The I-V characteristics displayed the various parameters applied by the solar module analyzer to detect and evaluate its performance. The curve fitting procedure of the I-V measurements was taken under a wide range of irradiance values experienced in the actual environment. The module displayed consistent performance and operated within the verified range. Several factors influenced the module during the testing procedure such as temperature variation, shading, irradiance, and mismatch losses. Such factors can have a significant effect on the module’s performance, affecting the overall harnessing of energy. Figure 3 provides the I-V curves representing the first comparison of the module’s parameters.

**Figure 4:**
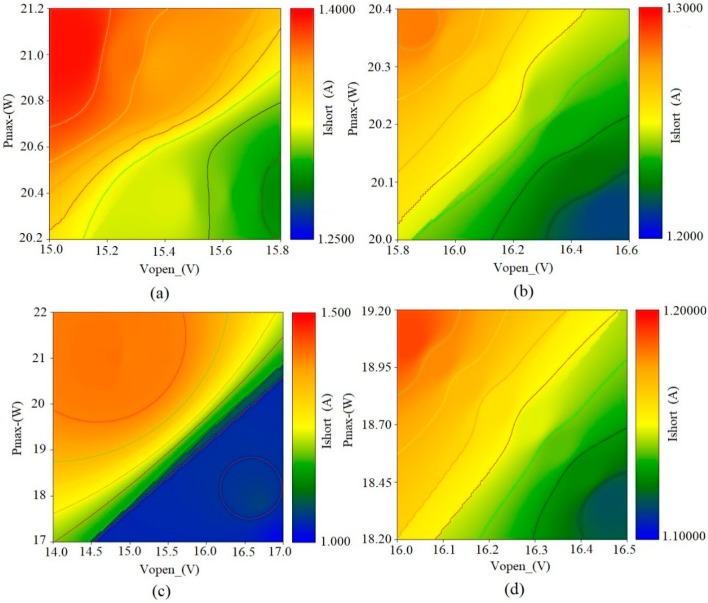
Tripartite analysis of the electrical performance of the developed module: (**a**–**d**) random readings depicting the relationships between variables; Vopen-(V), Ishort-(A) and Pmax-(W).

I-V curves present sufficient analysis to determine the module’s behavior under various weather conditions. The module achieved its target according to specific solar radiation and ambient temperature values by providing a higher Pmax. In the first phase (IV-a and IV-b), in IV-a; the module achieved a Pmax of 20.90 W and Vopen of 19.71 V. The Vmaxp reached 15.16 V and Imaxp was 1.378 A. In the IV-b curve, a higher Pmax value of 21.04 W was observed. The curve exhibited a Vopen of 19.59 V, Imaxp of 1.394 and Vmaxp of 15.08 V. The IV-a and IV-b curves exhibited an Ishort of 1.581 A and 1.597 A; respectively. The module’s performance factor (PF) compares its actual performance with theoretically designed specifications. Since the module was designed to achieve 20 W Pmax (even reaching 21.04 W Pmax), it proved to properly function against its theoretical characteristics and performance expectations. The validity of the theoretical interpretation for temperature was based on the basic I-V characteristics of the solar module which employs high purity polycrystalline (poly-Si) solar cells. The module effectively responded to two main factors in its overall performance: weather conditions and temperature. Reliable encapsulation is key for determining consistent efficiency measurements [22]. The efficiency of a solar module is directly linked to designed parameters. A change in solar parameters will cause a significant change in the evaluation of efficiency. Two major issues are encountered in photovoltaic (PV) systems: less conversion efficiency and reliance on weather conditions. The solar cells’ I-V characteristics can be non-linear due to the existing complex relationship between current and voltage as well as the prevailing variation of insolation or temperature fluctuation. The maximum power point (MPP) where the system provides the highest possible efficiency and produces maximum output power is a single point on the I-V characteristics curve. Failure to track the MPP is the main cause of power loss. It is essential to diligently monitor the MMP to ensure proper functionality [23]. The module displayed consistent I-V performance in the second phase (IV-c and IV-d) of the evaluation, with a variation of 0.78 W Pmax between the two curves. In the IV-c, the module achieved a Pmax of 20.01 W and reached 20.07 for Vopen. Such performance proved the high reliance on current and voltage achievements, especially in defining the module’s consistency and accuracy. In IV-d curve, the module’s performance slightly fluctuated due to changes in weather conditions, achieving 19.23 W for Pmax and 19.51 V for Vopen. The same evaluation set achieved a Vmaxp of 15.42 V and 15.20 V, Ishort of 1.452 A and 1.423 A and Imaxp of 1.297 A and 1.265 A. The experimental I-V characteristic curves demonstrated good electrical performance at different temperatures and irradiations. The comparison results revealed a high level of agreement between all testing phases, supporting the hypothesis of the module’s electrical dependability. The module’s efficiency naturally dropped when a change in weather occurred. This caused a reduction in radiation flow or heat escalation which affected the overall performance. The slight drop of efficiency is specifically attributed to the increase of surface temperature. Changes in temperature ranges are determined by various factors such as irradiance gain, weather conditions, outdoor temperature, and the module’s temperature. Solar module components and their characteristics are of paramount importance since they reveal a critical part of determining the module’s response to heat and heat dissipation. The ambient temperature was evaluated during the testing of the same phase evaluation. The module normally gains temperature when it is gradually or excessively exposed to high temperatures. A critical problem occurs when components raise the temperature of the solar module to the limit. This makes it deficient and reduces its performance. It is vital to investigate the properties of used components to understand the heat gain and heat transfer of glass, solar cells, encapsulations, and backsheets. Designing solar modules based on proper material components that demonstrate high thermal stability with functionally graded materials (FGMs) will establish enhanced heat management. This will ensure optimized energy harnessing [8,24]. The module achieved consistent performance in the final evaluation phase. Pmax reached 19.50 W and 20.39 W in the IV-e and IV-f assessments. Vopen reached 19.67 V and 20.24 V; respectively. Both curves exhibited an Ishort of 1.471 A, 1.467 A, Imaxp of 1.293 A and 1.310 A and Vmaxp of 15.08 V and 15.56 V; respectively. The module accomplished the target and was fit to perform according to the given parameters. Humidity and tropical rain did affect its overall performance. Tropical humid conditions, increased relative humidity, and high air temperature are known to affect electrical power generation [25]. Figure 4 provides the contour plots analyses including three variables: the maximum solar module power (Pmax), open-circuit voltage (Vopen), and short circuit current (Ishort). The tripartite analysis provided further clarification on the electrical performance of the developed module.

During the testing procedure, the tripartite analysis provided partial evaluation of the electrical performance of the developed solar module, considering the fluctuating weather conditions such as tropical rain, humid weather, cloudy weather, and solar spectrum intensity. The module exhibited stable performance and efficiently presented a tripartite analysis within the rate of acceptance. The variation that occurred between variables was considered to be natural since the module achieved better than its theoretical configurations. The correlations between the three variables are clearly presented. Each plot of the tripartite analysis consisted of batch measurements for equal numerical inputs derived from the three identified variables. In plot (a), the achieved Pmax was between 20.2 and 21.2, with Ishort between 1.250 and 1.4000. Vopen was between 15 and 15.8. Plot (b) displayed a slight decrease in Pmax, reaching 20.4, and Vopen was at 16.6. Ishort was between 1.2000 and 1.3000. The module demonstrated better performance in plot (c) achieving 22 Pmax and 17.0 Vopen. In this batch, Ishort was between 1.000 and 1.500. The final plot (d) showed Pmax between 18.20 and 19.20. Vopen was between 16.0 and 16.5, with Ishort ranging between 1.1000 and 1.2000. The tripartite analysis considered three main variables that provided further understanding on module’s efficiency. The variables were the open-circuit voltage (Vopen), short-circuit current (Ishort) and maximum electric power (Pmax). They represent the VIP analysis where the correlations between the three variables can be simultaneously addressed. In the given tripartite analysis, semi-positive correlations can be observed between the three variables and this can be considered as normal since the performance of the module is observed to be consistent. However, the three variables are not only important to determine the maximum electrical power and efficiency [26], they are also essential for determining the fill factor (FF), since the performance of a solar module at real conditions relies on the varying metrological conditions. The solar irradiance and module’s temperature affect the open-circuit voltage, short-circuit current and overall output. Hence, it is important to evaluate these parametric values to further comprehend the emerging relationships [27].

It is necessary to evaluate weather conditions, ambient humidity, temperature, bias voltage, and current leakage when modelling PV performance. Investigating the impacts of thermal characteristics of solar cells, encapsulation films, and their respective backsheets are also necessary [28]. The electrical performance of the module was mainly subjected to changing tropical weather conditions. Thermal performance was evaluated accordingly. The module presented evidence of improved thermal stability attributed to PVDF-SSPF composites. The efficiency of solar cells and the unavoidable temperature impact were additional reasons behind the slight fluctuations observed in the module’s performance during the outdoor evaluation. Relative humidity, wind speed, and dust concentration can affect a solar module’s power generation and overall performance. Such factors cause surface soiling, leading to an evidenced drop in performance. The ambient environmental analysis is crucial for conducting accurate system simulations [29]. The modules are highly influenced by weather parameters. A rise in air temperature and relative humidity can cause a reduction in the intensity of solar radiation. Weather conditions influence solar module voltage and power production variations. It is imperative to distinguish the system response due to these variations. This can eventually contribute to the proper design and fabrication of solar modules [30]. Field test results demonstrated the actual evaluation model that can accurately predict I-V curve characteristics to further understand the electrical behavior. Table 1 provides a summary of information on the achievements of the three evaluation phases of the developed solar module.

### 3.2. Thermal Performance of Solar Module

**Figure 5:**
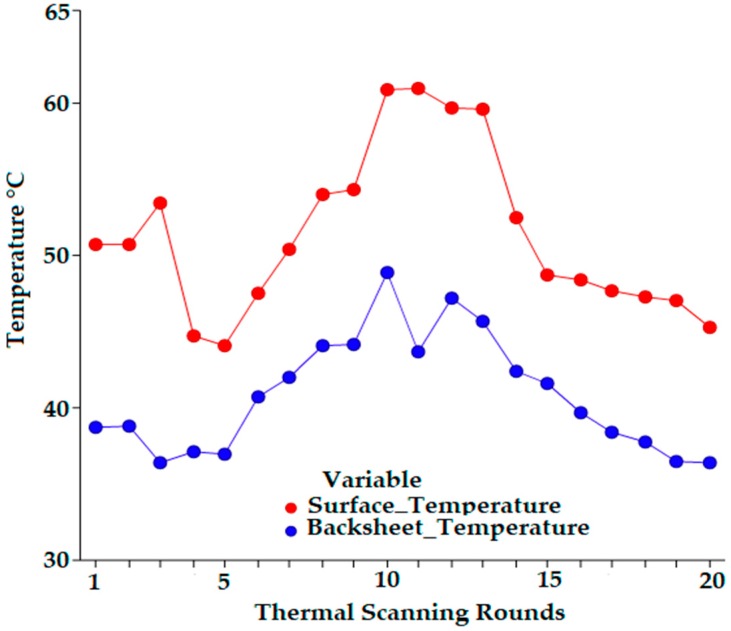
Thermal behavior of the developed solar module representing the performance of the surface and backsheets.

In the first thermal analysis, 20 consecutive readings were made to ensure consistency and accuracy in the thermal behavior evaluation. Throughout the entire evaluation process, the thermal analysis was accomplished to investigate the thermal performance of the surface and PVDF-SSPF composites. Figure 5 presents the thermal relationship between the surface temperature and the PVDF-SSPF backsheets in the developed module.

**Figure 6:**
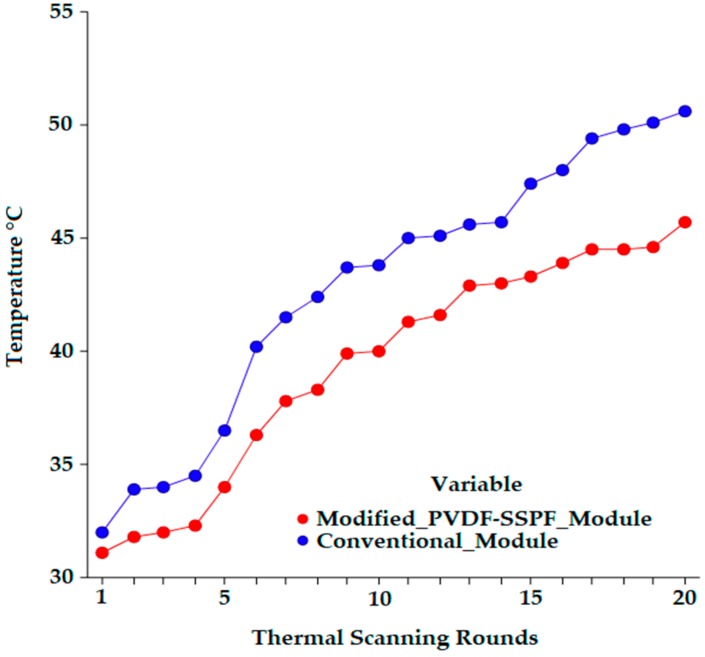
Proportional performance of the thermal behavior of backsheets attached to the tested modules.

The surface temperature was reported to uphold higher levels in all cases of direct exposure to sun radiation. The average temperature of the backsheet compared to the surface temperature exhibited enhanced thermal performance. The average surface temperature was 51.39 °C while the average backsheet temperature was 40.86 °C. The total average variation between the two temperatures was 10.53 °C. The surface temperature of the solar module was higher compared to the backsheet temperature. This is due to the direct exposure of sun radiation and heat on the solar surface. The backsheet was located at the back side of the solar module. Less heat should be observed. Significant determinants of the backsheet’s thermal behavior include two key factors: the gap range between the surface temperature and backsheet, and the consistency of maintaining lower temperatures in the backsheet in contrast to increasing temperatures of the module’s surface. These factors can be achieved by assessing the proportional thermal performance of surface and backsheet temperatures. Some designed backsheets may be slower in receiving heat from the surface temperature or weather conditions due to their thermal characteristics. Heat may take a longer time to dispatch. The operating temperature or the solar cell temperature can be influenced by specific variables and measurements such as meteorological variables: ambient temperature, irradiance, wind speed, and direction. Electrical operations, such as maximum power point and open circuit, play a key role as well as installation procedures such as building integration and shed. The temperature coefficients (including short circuit, open circuit voltage, and maximum power thermal coefficient) influence the conversion efficiency of photovoltaic (PV) systems. Complexity rises when evaluating such factors. PV modules encounter efficiency problems during high irradiance records. Various mechanisms are employed to determine the development of heat in a solar module such as the joule effect on the internal parasite resistance and the partial transformation of photon energy into heat. Such phenomena increase temperatures, consequently causing a reduction of the module’s conversion efficiency [31]. The surface and backsheet performance of the developed module displayed evidence of thermal stability and excellent proportional performance in tropical and humid climates. This may be attributed to the utilization of PVDF-SSPF composites. They possess satisfactory thermal and optical features with the use of natural fiber composites (NFCs) [6,7]. A comparative thermal experiment was conducted to investigate the thermal behavior of PVDF-SSPF and conventional backsheets. Both modules were configured using similar parameters and tested under exact weather conditions. Figure 6 provides the thermal behavior of the attached backsheets.

**Figure 7:**
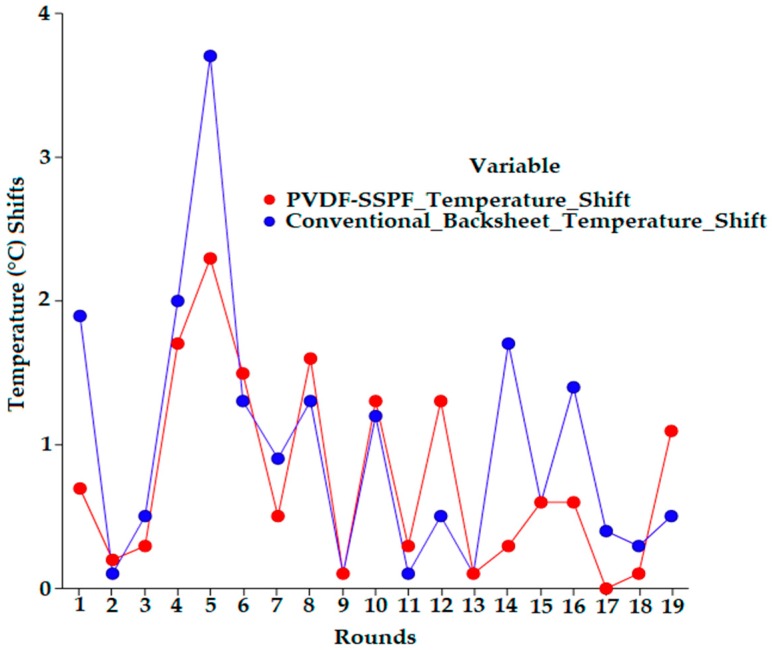
Temperature shifts of PVDF-SSPF and conventional backsheets.

The backsheets of the attached modules responded to increased heat and high radiation. The responsiveness of both backsheets varied since different readings were observed. The PVDF-SSPF backsheet exhibited less temperature absorbance and higher thermal stability compared to the conventional backsheet. During the test, 20 uninterrupted readings were simultaneously taken. The initial readings were 31.1 °C and 32 °C for PVDF-SSPF and conventional backsheets. Final readings were 45.7 °C and 50.6 °C. When the ambient temperature increased and the radiation exhibited higher values, both backsheets gradually responded to the change in temperature. The conventional backsheet responded faster than the PVDF-SSPF backsheet, signifying a higher acceleration in temperature. This temperature increase can be detrimental since it will eventually affect the performance of the utilized solar cells. Figure 7 provides information on the temperature shifts. A comparison was made between PVDF-SSPF and conventional backsheets.

**Figure 8:**
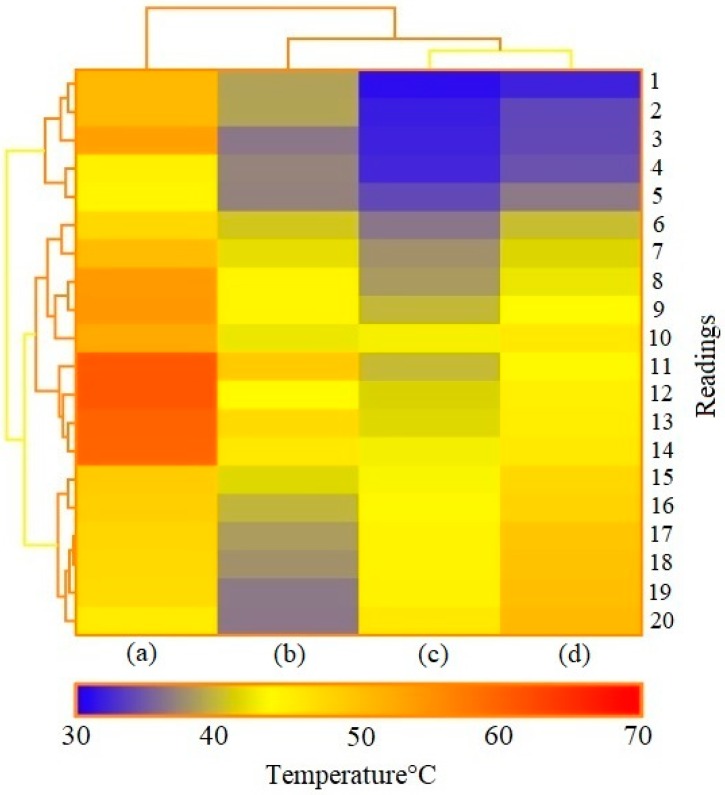
Heatmap analysis showing the correlations and thermal performance of (**a**,**b**) surface temperature of the developed module and backsheet temperature of the developed module in a simultaneous test; (**c**,**d**) PVDF-SSPF backsheet temperature and conventional backsheet temperature under similar weather condition test.

PVDF-SSPF performed adequately with an average variation of 3.52 °C. This represents 8.54% of the total average of the reported temperature in both tests. The gradual change of temperature was under constant observation. The conventional backsheet displayed a rapid shift in temperature. Readings for the conventional backsheet were 36.5 °C, followed by 40.2 °C, with a shift of 3.7 °C. Readings for PVDF-SSPF backsheets were 34 °C, followed by 36.3 °C, with a shift of 2.3 °C. When temperatures were 43 °C and 43.9 °C, with a shift of 0.9 °C in the fabricated composite, readings were 45.7 °C and 48 °C, with a shift of 2.3 °C in the conventional one. The total shifts between PVDF-SSPF and conventional backsheets were 14.6 °C and 18.6 °C. The obtained results provide evidence of enhanced thermal stability and higher efficiency in the developed module. The proportional performance proved that the introduced composite was thermally stable and supported heat reduction in solar modules. Temperature has a strong influence on the module’s electrical performance. Such performance can be improved by reducing escalating temperatures. Current solar cells cannot convert all energy into electricity. As the temperature of the solar cell increases while exposed to sun radiation, the band gap of the module decreases. Like all semiconductor devices, solar cells are sensitive to temperature. Increase in the band gap leads to an increase of the short circuit current and a decrease of the open circuit voltage. This occurs due to the existence of thermally excited electrons which dominate the electrical functions of silicon cells [32]. The PVDF-SSPF backsheet exhibited good encapsulation and adhesion strength, which makes it fit to serve as a backsheet for solar/PV modules. The structure was comprised of PVDF reinforced with SSPF and bound with solar cells. The conventional backsheet consisted of encapsulated polymeric materials and laminates adhesively bound to each other. Both backsheets proved to adequately function despite the thermal and technical variations in their performance. Favorable encapsulation and backsheet properties contribute to the module’s reliability and lifetime performance. The degradation of adhesive strength between the backsheet layers and encapsulants may cause a failure mode in the PV module [33]. The mechanical, optical, electrical, and chemical properties of solar backsheets are critical for long-term reliability, safety, and durability of solar modules. The backsheets form the outer and protective layers of solar panels. They are key factors in the performance and durability of a solar module. Polyester based backsheets can degrade, crack, and display a change in color. Yellowing indicates degradation and change in backsheet properties which influence the module’s performance. Degradation, yellowing, and cracking of commercial backsheets’ inner and outer layers can cause common failure in solar module mechanisms [34]. This necessitates further investigations for more adequate materials of solar module backsheets to enhance their overall performance. Figure 8 provides a heatmap of the thermal analysis, showing the correlations and thermal performance of surface-backsheet temperature analysis, thermal analysis of the backsheet in the developed module, and thermal analysis of the backsheet in the conventional module.

Heat dissipation is one crucial factor that contributes to higher efficiency in solar modules and prevents thermal degradation of their components [35]. In the given heatmap, PVDF-SSPF maintained better thermal stability and was more consistent in responding to increasing temperatures. This could be attributed to the thermal properties of PVDF since the polymer is thermally stable to be utilized in nano, complex, and advanced materials [36,37]. Natural fibers are also are known to be excellent materials with specific thermal stability [38,39,40]. In this work, untreated SSPF was utilized since it was proven that untreated SPF is significantly more stable than treated fiber [41]. The (b) and (c) columns provide evidence of excellent thermal performance exhibited by the PVDF-SSPF backsheets. Column (c) provided much better thermal stability and showed less heat absorbance compared to conventional backsheets. To further understand the responsiveness of used materials, PVDF falls under fluoropolymers and consists of fluorine atoms in its structural formula. The thermoplastic polymer exhibits excellent thermal stability with outstanding mechanical and physical properties [5,42]. SSPF is a natural component and an alternative to synthetic fibers. It is known for its outstanding thermal, physical, and mechanical properties such as good tensile strength and water resistance [43,44]. In PVDF-SSPF composites, both materials achieved better thermal stability. This can be attributed to the good interfacial bonding observed, as well as the homogenous structure of composites. Table 2 provides a summary of information on the proportional thermal performance of PVDF-SSPF and conventional backsheets attached to the tested solar modules.

## 4. Conclusions

This work features the development and electrical-thermal evaluation of a solar module encompassing PVDF-SSPF backsheet composites. Electrical and thermal performances were evaluated. The thermal performance of backsheets was assessed and compared with conventional backsheets. It is concluded that:

- The electrical assessment provided evidence of reasonable electrical efficiency by achieving satisfactory I-V characteristics. The developed module attained the Pmax range of 19.23 W to 21.04 W and Imaxp range of 1.265 A to 1.394 A. Vopen was between 19.59 V and 20.24 V;
- The backsheet temperature exhibited adequate thermal stability in correlation to the surface temperature. The total average variation between the two temperatures was 10.53 °C. This was attributed to the consistency of maintaining lower temperature in the backsheet compared to increasing temperatures of the module’s surface, as well as the gap range between the surface temperature and backsheet placement;
- In the proportional analysis between PVDF-SSPF and the conventional backsheet, both backsheets responded differently to the change of temperature and ambient climate. PVDF-SSPF was less responsive to temperature and heat absorbance. The start-end points for PVDF-SSPF and conventional backsheets are (31.1 °C, 45.7 °C) and (32.0 °C, 50.6 °C);
- The temperature shifts verified the improvement in thermal stability and the reduction in heat absorbance in PVDF-SSPF backsheet composites. The average variation was 3.52 °C which represents 8.54% of the total average of reported temperatures in both tests. The total temperature shift was determined as 14.6 °C for PVDF-SSPF backsheet composites and 18.6 °C for the conventional one;
- This work recommends additional research efforts to investigate the characteristics of the developed module. Further analyses are needed to demonstrate functional characterizations. These can be accomplished using accelerated thermal-endurance and degradation testing, damp heat testing, heat dissipation analysis, and cumulative thermal modeling.

## 5. Patents

This work is patent pending.

## Figures and Tables

**Table 1:** A summary of verified achievements of three evaluation phases of the developed module.

Phase	Readings	Temperature (°C)	Vopen (V)	Pmax (W)	Vmaxp (V)	Ishort (A)
**A1**	**IV-a**	28–33	19.71	20.90	15.16	1.581
	**IV-b**	28–33	19.59	21.04	15.08	1.597
**A2**	**IV-c**	28–33	20.07	20.01	15.42	1.452
	**IV-d**	28–33	19.51	19.23	15.20	1.423
**A3**	**IV-e**	28–33	19.67	19.50	15.08	1.471
	**IV-f**	28–33	20.24	20.39	15.56	1.467

**Table 2:** A summary of thermal readings based on temperature (°C) of PVDF-SSPF and conventional backsheets.

Type	Temperature °C									
PVDF-SSPF	31.1	31.8	32	32.3	34	36.3	37.8	38.3	39.9	40
Conventional	32	33.9	34	34.5	36.5	40.2	41.5	42.4	43.7	43.8
PVDF-SSPF	41.3	41.6	42.9	43	43.3	43.9	44.5	44.5	44.6	45.7
Conventional	45	45.1	45.6	45.7	47.4	48	49.4	49.8	50.1	50.6
